# Status and Dietary Intake of Phytoene and Phytofluene in Spanish Adults and the Effect of a Four-Week Dietary Intervention with Lutein-Rich Fruits or Vegetables

**DOI:** 10.3390/nu14142922

**Published:** 2022-07-17

**Authors:** Elena Rodríguez-Rodríguez, Rocío Estévez-Santiago, Milagros Sánchez-Prieto, Begoña Olmedilla-Alonso

**Affiliations:** 1 Department of Chemistry in Pharmaceutical Sciences, Faculty of Pharmacy, Complutense University of Madrid (UCM), 28040 Madrid, Spain; 2 Facultad de Ciencias de la Salud, Universidad Francisco de Vitoria (UFV), 28223 Madrid, Spain; rocio.estevez@ufv.es; 3 Department of Metabolism and Nutrition, Institute of Food Science, Technology and Nutrition (ICTAN-CSIC), 28040 Madrid, Spain; msprieto@ictan.csic.es

**Keywords:** phytoene, phytofluene, dietary intake, status, humans, faeces

## Abstract

Phytoene (PT) and phytofluene (PTF) are colourless carotenoids presents in the human diet and in blood, faeces and tissues and are biologically active. However, there is very little data on these carotenoids. This study aims to assess PT and PTF concentrations in serum from healthy Spanish normolipemic subjects (*n* = 101, 45–65 years) and the effect of a fruit and vegetable dietary intervention (4 weeks, *n* = 29) on PT and PTF concentration in serum and faeces and dietary intake. Serum and faecal concentrations were analysed by HPLC and dietary intake by 3 × 24 h recalls. PT showed higher concentrations than PTF in serum, faeces and in the dietary intake. Considering both studies, PT and PTF concentrations in serum were 0.16 ± 0.07 and 0.05 ± 0.04 µmol/L, respectively, in faeces 17.7 ± 20.3 and 6.5 ± 7.9 µg/g, respectively, and in dietary intake the median was 2.4 and 0.6 mg/p/day, respectively. Carrots and tomatoes were the major dietary contributors of these carotenoids. The dietary intervention did not cause significant variations in the PT and PTF intake or serum concentrations, but a lower concentration in faeces was observed for the fruit group (PT: *p* = 0.024; PTF isomer-3: *p* = 0.034). These data highlight the need for further research on the activities of these carotenoids in humans.

## 1. Introduction

Phytoene (PT) and phytofluene (PTF) are C40 colourless carotenoids with short chains of conjugated double bonds (3 and 5, respectively). They are known to be universal precursors in the biosynthesis of coloured carotenoids in photosynthesizing organisms and are therefore likely present in a large array of carotenoid-containing foods [1]. The presence of PT and PTF has been reported in a variety of fruits and vegetables and can be found in products such as tomatoes and their derivatives, apricots, red peppers, carrots oranges or red grapefruits [1,2]. These carotenoids were present in about one-third of the foods consumed by a representative sample of the Spanish adult population, carrots, apricots, tomatoes, and oranges being the major contributors [2]. Similarly, apricot and tomato ketchup were the highest contributors in a study conducted in Luxembourg [3]. In both populations, PT and PTF intakes were around 2 and 0.6 mg/p/day, respectively [2,3], comparable to the intake of the main dietary carotenoids habitually assessed [4].

PT and PTF have been detected in significant quantities in both human plasma and tissues, including liver, lung, breast, skin and prostate, where they may provide specific health benefits because of their antioxidant and anticarcinogenic activities [1,5,6]. Epidemiological studies indicate that they may help reduce the risk of developing certain types of cancer [7]. Typically, PT and PTF levels in plasma are in the 0.1–1.0 μM range, the former with slightly higher levels than the latter [7]. Concentrations of these carotenoids in plasma increased when PT and PTF-rich foods were ingested [8,9], while the restriction of such foods or the intake of foods with a low content in these carotenoids caused a decrease of their concentration in plasma, with PT decreasing faster than PTF [10].

These coluorless carotenoids are biologically very active, exhibiting anti-inflammatory, antioxidant and anticarcinogenic properties both in-vitro and in-vivo. For instance, these carotenoids can act either additively or synergistically with CoQ10 to lower the production of inflammatory mediators in UV-irradiated or IL-1 in human dermal fibroblast cell cultures and they can protect CoQ10 from degradation [11]. PTF and its oxidation products induced apoptosis in HL-60 cells as a cancer cell model after 24 h incubation, similar to lycopene and its oxidation products [12], and PT inhibited cancer cell proliferation induced by 17-β-estradiol and genistein [13]. Regarding antioxidant activity, a PT-enriched tomato fraction (also containing PTF) had an antioxidant response in-vitro [14] and this carotenoid also reduced the production of intracellular reactive oxygen species in HepG2 cells. In line with what was observed in the in-vitro studies, in a double-blind cross-over study, 26 healthy subjects who consumed 250 mL of a tomato drink (providing about 4 mg PT, 3 mg PTF, and other carotenoids) for 26 days exhibited a reduction (by about 42%) in DNA damage to lymphocytes subjected to oxidative stress, thus showing that these carotenoids have an antioxidant effect [15]. However, despite these biological activities and the fact that PT and PTF intake is comparable to and sometimes even higher than that of the other carotenoids typically included in human health studies, there is still very little data on their content in foods [15,16] and even less data on their concentrations in blood, faeces and tissues [17,18].

With an aim to gather data on these colourless carotenoids, our objectives were to assess the PT and PTF nutritional status and dietary intake in a group of healthy Spanish adults between the ages of 45 and 65, and to evaluate the effect that a dietary intake intervention with fruits or vegetables had on that status.

## 2. Material and Methods

### 2.1. Subjects

101 participants (77 women, 24 men), aged 45 to 65 years (mean ± SD: 54.4 ± 0.6 years), enrolled in a previous observational study to assess lutein and zeaxanthin dietary and status markers [19]. Briefly, the inclusion criteria were normal cholesterolemia (upper limit ca 6.22 mmol/L), body mass index ≥20 and ≤30 kg/m^2^, mixed diet and the following exclusion criteria: consumption of dietary supplements, use of drugs or phytosterol-enriched beverages/foods to control cholesterolaemia and chronic diseases that can affect carotenoid or lipid metabolism. Two subgroups of those volunteers participated in a four-week dietary intervention study with fruit (*n* = 14) or vegetables (*n* = 15) rich in lutein [20]. The foods supplied in the intervention study were as follows: (a) fruits (500 g/day): avocados (250 g), kiwi (100 g), oranges (150 g); (b) vegetables (180 g/day): green beans (110 g), pumpkin (50 g) and sweet corn (20 g). The intake of lamb’s lettuce (18–20 g/day) was included in both groups (fruit and vegetables). These foods were selected to supply 1.8 mg lutein /day in each group [20]. These foods supplied 0.09 and 0.07 mg/d of PT and PTF, respectively, in the fruit group and 0.11 and 0.05 mg/day of PT and PTF, respectively, in the vegetable group.

The present study included samples from participants in those previous studies (observational and intervention). Serum samples were analysed to evaluate PT and PTF concentrations in these two studies. Food records involving 24 h recalls (×3) and faeces samples were analysed in the fruit and vegetable intervention study.

The Ethical Committee of Research with Drugs of the Hospital Universitario Puerta de Hierro Majadahonda of Madrid, Spain (13 February 2017, acta *n* 03.17) and the CSIC’s Ethics Committee, Bioethic Subcommittee (21 February 2017) approved the study. An informed consent was signed by all subjects.

### 2.2. Carotenoid Extraction and Analysis in Blood, Faeces and Food Sample

Carotenoids extraction in serum was performed using ethanol and hexane: dichloromethane (5:1) stabilized with 0.1 g/L butylated hydroxytoluene (BHT), following a previously described process [19,20]. Once the extract was dried, it was reconstituted with methanol (MeOH): methyl-ter-butyl ether (MTBE) (1:1) and injected into the HPLC system.

To extract carotenoids in faeces, each lyophilized sample was homogenized with Phosphate-Buffered Saline (PBS), ethanol and acetone and magnetically stirred. Supernatant liquid was then removed and diethyl ether (DE): petroleum ether (PE) was added, stirred and centrifuged, repeating the process until colourless. The extract was then dried and dissolved in MeOH: MTBE (1:1), filtered and kept at −20 °C until analysis, as previously described [20].

Only two of the foods included in the intervention studies were analysed for PT and PTF content—orange and pumpkin; content in other foods ingested by the participants was obtained from the literature [2,21]. A previously described extraction procedure was followed [22]. Briefly, acetone, celite and ethanol were used for the extraction and the process was repeated until colourless. Thus, an L/L was used with DE:EP (1:1), according to a previously described protocol [22]. The extract was dried, reconstituted in MeOH: MTBE (1:1) and filtered. It was then injected into the chromatographer.

### 2.3. HPLC-DAD Carotenoid Analysis

Carotenoid concentrations were determined by high performance liquid chromatography (HPLC) using a system consisting of a model 600 pump, a Rheodyne injector and a 2998 photodiode array (PDA) detector (Waters, Milford, MA, USA) and a C30 YMC column (5 µm, 250 × 4.6 mm i.d.) (Waters, Wilmington, MA, USA) with a guard column (Aquapore ODS type RP-18) at room temperature (25 °C). Mobile phase was formed by MeOH with 0.1% trimethylamine (solvent A) and MTBE (solvent B) in a linear gradient. At baseline, 25, 55 and 60 min the ratios of the solvents were 95:5, 70:30, 35:65 and 95:5. The detection was performed at 285 nm for both PT and tocopheryl acetate (internal standard) and 270 nm for PTF. Chromatograms were processed using Empower 2 software (Waters, Milford, MA, USA).

**Figure 1:**
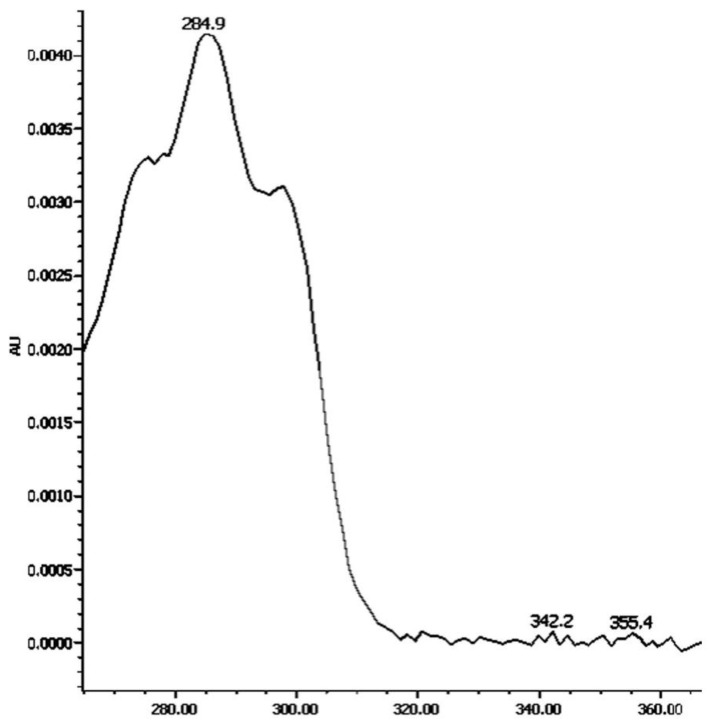
UV-spectra of phytoene at 285 nm.

Identification of PT was based on the retention time (RT) of standard solution (16.4 min) and comparison with absorption spectra reported in the literature (276, 286, 300 nm) [23]. Serum PT was quantified using a calibration curve at three concentration levels (15, 30 and 50 ng/μL (y = 8.18 × 10^3^ × −2.76 × 10^4^; R^2^ = 0.994). PT in faeces was calculated by means of a response factor against a PT standard solution of 12.5 ng/μL (Figure 1).

No standard was available for PTF, so it was tentatively identified by comparing the RT and spectra obtained with those described in the literature and quantified against a PT standard (by response factor for faeces and a calibration curve for serum samples).

### 2.4. Phytoene and Phytofluene Dietary Intake Assessment

Three 24-h diet recalls were conducted within a period of seven days, one of which coincided with a weekend or holiday, to assess the PT and PTF dietary intake. Foods and amounts consumed (g edible portion/day) were introduced into a specific software application for carotenoid intake calculation [24] in which PT and PTF content in 66 foods is included (33 fruits, 28 vegetables and 5 juices and processed foods) [2].

### 2.5. Statistics

Data are expressed as the mean ± standard deviation (SD) and median. The Kolmogorov–Smirnoff test was used to check whether variables were distributed normally. Most of the variables featured a normal distribution and those that did not, were Ln transformed. Student’s *t* test for paired data was used to assess the significance of changes within each intervention group, while the *t*-Student test was used to assess the differences between the intervention groups. All reported *p*-values were based on a two-sided test and compared to a significance level of 0.05. SPSS Statistics Editor (IBM SPSS Statistics, v.27) was used for all statistical calculations.

## 3. Results

### 3.1. Identification of PT and PTF by HPLC

**Figure 2:**
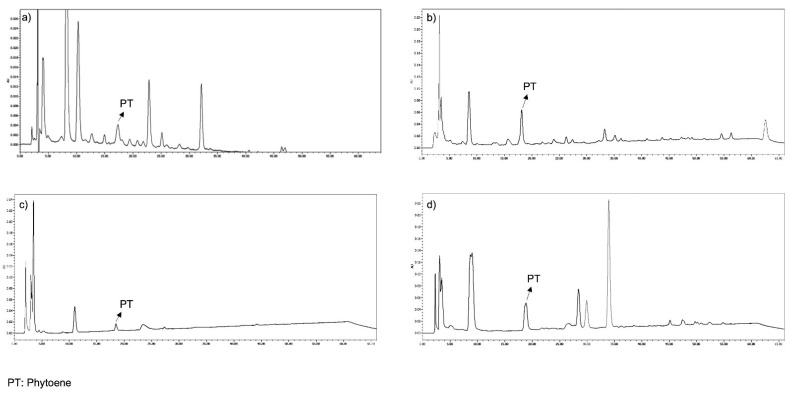
Chromatograms at 285 nm of a sample of serum (**a**), faeces (**b**), orange (**c**) and pumpkin (**d**).

**Figure 3:**
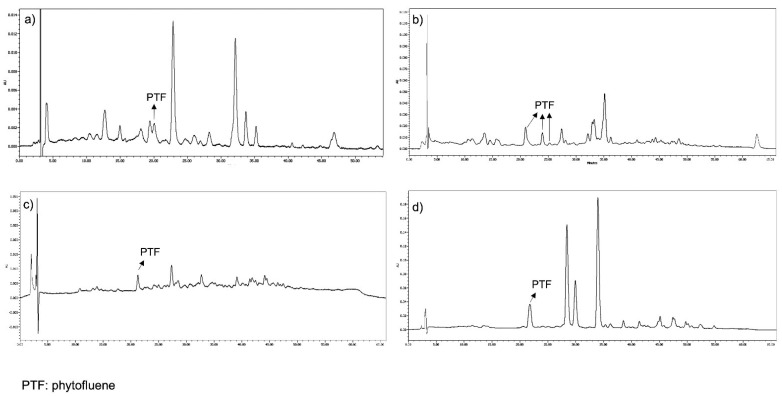
Chromatogram at 370 nm of a sample of serum (**a**), faeces (**b**), orange (**c**) and pumpkin (**d**).

PT and PTF were well separated in the chromatograms from the three types of samples extracted: serum, faeces and foods. They were detected at 285 and 370 nm, respectively, and eluted at approximately 17.5–18.7 and 19–23.5 min, respectively. PT eluted as a single peak in all the samples analysed (Figure 2), and its spectrum showed three absorption peaks in all the samples analysed, the highest at 284.9 nm. In serum and food, PTF eluted as a single peak with absorption peaks at 330, 347 and 363.5 nm. However, in faeces, PTF eluted as three peaks and exhibited the same absorption peaks just mentioned and with retention times of 19.5, 22.5 and 23.5 min (Figure 3).

### 3.2. PT Dietary Intake

The major dietary contributors of PT and PTF in the observational study and at baseline in the intervention studies were carrot and tomato, respectively (Table 1 and Table 2). At the end of the intervention study, the contributors were the same but in reverse order: tomato and carrot (Table 2).

No statistically significant differences were found in PT and PTF dietary intake among the participants in the observational study or the fruit and vegetable dietary intervention groups at baseline (Table 1 and Table 2).

Of the foods supplied in the intervention study, the only ones that contained PT and PTF were oranges and pumpkin. Specifically, oranges had a 73.0 and 33.3 µg/100 g PT and PTF content, respectively, and pumpkin 185.9 and 144.84 µg/100 g.

In the intervention group, the consumption of carrots in the fruit group dropped significantly (from 16.1 ± 29.7 to 6.6 ± 11.1 g/day), as did that of orange juice and fried tomato in the vegetable group (from 24.5 ± 48.0 to 0 g/day and from 9.6 ± 15.3 to 0.75 ± 2.91 g/day, respectively). The four-week fruit and vegetable dietary intervention did not cause significant variations in PT or PTF intake in any of the groups. Only the PT and PTF contributed by carrot decreased in the fruit group and the PT and PTF from orange juice and the PTF from fried tomato in the vegetable group. In intervention subjects whose PT and PTF intake increased or remained stable, changes in their serum and faeces concentrations were also assessed, and no significant difference was observed.

### 3.3. PT and PTF Serum Concentrations

The mean PT and PTF concentration in the serum of subjects in the observational study and in the intervention study at baseline were 0.158 ± 0.067 and 0.054 ± 0.037 µmol/L, respectively. The serum values for each group are shown in Table 1 and Table 2. No significant differences in PT or PTF levels were observed between the groups at baseline or at the end of the intervention study. At the end of the 4-week intervention period, no changes were observed in PT or PTF concentration of serums in either intervention group.

### 3.4. PT and PTF Concentrations in Faeces

PT and PTF were analysed in faeces samples from the participants in the fruit and vegetable intervention study. Faeces data from the observational study has been published elsewhere [18]. PT concentration in faeces at baseline did not show differences between the two intervention groups (fruit and vegetable). In the fruit intervention group, PT concentration in faeces decreased significantly. However, at the individual level, this decrease was only observed in participants who, before the intervention, had the lowest PT values in serum, i.e., less than p50 ≤ 0.107 µM (in these individuals it decreased in faeces from 22.02 ± 8.79 to 12.06 ± 5.69 μg/g (dried weight), with no variation in serum (0.092 ± 0.010 µol/L at the beginning and 0.094 ± 0.015 µmol/L at the end of the intervention) or in diet (2521.6 ± 2761.0 at the beginning and 1645.5 ± 1496.7 μg/day at the end).

The PTF concentration in faeces at baseline did not vary between the two intervention groups either. In the intervention group, there was a decrease in PTF isomer 3 of the fruit group, which was smaller than in the vegetable group (Table 2). A more detailed analysis showed that the decrease in PTF 3 only occurred in those individuals of the fruit group that initially had a low PTF concentration in serum, i.e., lower than the p75 value for that group (0.060 µmol/L). In these individuals, PTF 3 decreased from 0.82 ± 0.52 to 0.49 ± 0.29 μg/g (with no change in dietary or serum values between the beginning and the end of the intervention, these latter two values being 578.0 ± 540.3 vs. 494.2 ± 328.7 μg/day and 0.025 ± 0.014 vs. 0.027 ± 0.010 µmol/L, respectively).

## 4. Discussion

PT and PTF are carotenoids present in the human diet and biological tissues which have received very little attention compared to other carotenoids mainly present in human blood whose relationship between diet and nutritional status has been widely researched [25]. This study sheds light on these carotenoids by providing data on their dietary intake and concentration in blood and in faeces in a well-defined group of subjects (apparently healthy, normolipemic adults aged 45–65) who do not take food supplements.

The PT and PTF dietary intake in this study was similar to that described in a representative sample of the Spanish [2] and Luxembourg population [3], PT intake being greater than PTF. This higher intake of PT coincides with its higher prevalence in foods [2,7]. In these studies (observational and intervention studies at baseline), the dietary intake of PT and PTF was 2.8 mg and 0.7 mg, respectively. This PT intake was higher than that described in the Spanish (1.9 mg PT) and Luxembourgian (2 mg PT) populations, and PTF was similar to the Luxembourgian (0.7 mg PTF) but higher than the Spanish (0.5 mg PTF) population. Several different factors need to be taken into account when comparing intake among different population groups such as concentration in foods, use of different dietary intake assessment methods [26] and the age of the participants [27]. PT and PTF concentration in the foods assessed in this study were the same as those used in the study in a representative Spanish population, with the exception of oranges and pumpkin that were analysed for this study. However, these data do not coincide with those considered in the Luxembourg study, and these different concentrations in foods can lead to major differences in dietary intake. Also, there are many factors affecting the carotenoid content in foods, even foods of the same variety [25,26]. For example, in a study using the same variety of tomato, grown in the same place, at the same time and under the same conditions, major differences were found in PT and PTF content (6 and 4-fold, respectively) due to different degrees of ripeness and related to the expression of the phytoene synthase-1 gene (Psy-1) [27]. The dietary intake assessment method used in this study was similar to that used in the Spanish population study (food consumption registers) but different from the method used in the Luxembourg study (self-administered frequency questionnaire). The age of the participants is also relevant, as people aged 45–65 consume more fruits and vegetables compared to younger Spaniards [28]. The age range in the Spanish and Luxembourg populations studies was 18–64 and 18–69.

When comparing the PT and PTF intakes (2.8 and 0.7 mg/p/day) with that of the six main carotenoids typically assessed in health and diet studies, in the Spanish population PT intake (2.8 mg/p/day) was higher than that described for zeaxanthin (0.06 mg/p/day), α-carotene (0.27 mg/p/day), β-cryptoxanthin (0.32 mg/p/day) lutein (0.70 mg/p/day) and β-carotene (1.56 mg/p/day) [29,30]. PTF intake (0.7 mg/p/day) was also higher than most of these, except for β-carotene and lycopene. A comparison or our data with the average daily intake of other carotenoids as found in 23 studies around the world recently compiled by Böhm et al. [4], including the aforementioned in Luxembourg study, shows the intake of PT to be below that of β-carotene (4.1 mg/p/day) and lycopene (4.6 mg/p/day), while the intake of PTF, in addition to being below that of β-carotene and lycopene, is also below lutein/zeaxanthin (2.2 mg/p/day),is similar to α-carotene (0.7 mg/p/day) and higher than β-cryptoxanthin (0.3 mg/p/day).

For the PT and PTF dietary intake assessment, concentrations of these compounds were taken from literature [2], except for the orange and pumpkin, foods included in the intervention study and analysed in this study. The chromatogram showed only a single peak for each of these carotenoids, possibly corresponding to the cis-isomer because, as already described, the 15-cis-PT form is considered to be the predominant isomer in most carotenogenic organisms [31] and the cis-form is predominant in foods [15,32,33]. There is also evidence that some PT and PTF cis-isomers are more stable than their all-trans forms [34]. However, it is worth mentioning that there is only one isomer in these foods, whereas most foods have several isomers, as is the case of apricot, tomato and carrot, in which all-trans and 15 cis-isomers of PT were detected: in sanguinello juice, however, only the 15 cis-isomer was found [15], which corresponds with our results.

Two peaks for PT have been reported for Dovyalis and Tamarillo fruits under similar chromatographic conditions (C30 column and a methanol/MTBE in linear gradient). These peaks eluted at 17.7–18.1 and 18.5 min, both with spectral peaks at 276, 286 and 300 nm. The geometrical isomer peak configuration could not be assigned due to the limitation of the PDA detector that was at 190 nm, making it impossible to observe the PT cis-peak [35]. In a different study, with the same column and with ammonium acetate/methanol/MTBE as mobile phase, two PT peaks were also detected in tomato-based sauces, the major one being identified as 15-Z-PT and the other as all-E-PT. Two major PTF peaks were also detected, the major one identified as the Z-isomer and the minor one as all-E-PTF [33].

In serum chromatograms, a single peak at 285 nm was identified as PT through a comparison of its retention time with a PT standard. This coincides with the Hsu et al. study [17] that identified a PT peak at 25.26 min with a spectra peak at 276, 286 and 300 nm using a C30 column and with a binary solvent system of methanol/acetonitrile/water (84:14:4, v/v/v) and dichloromethane (100%). However, in a study designed to develop an HPLC method for the analysis of the highest number of geometrical isomers of the main dietary carotenoids found in humans, and using the same chromatographic conditions as in the present study (C30 column and methanol/t-MBE as mobile phase), no detectable levels of PT were observed in the plasma of two participants in a postprandial study with fruits and vegetables [36].

In serum chromatograms, a single peak at 19 min, with a maximum absorption at 370 nm, was observed and identified as PTF. However, several PTF isomers have been described in human serum, such as those reported by Hsu et al. [17] at 25.01 and 26.72 min, with spectra at 332, 348 and 366 nm (Z-PTF), and at 334, 350 and 370 nm, respectively. In the above-mentioned Melendez–Martínez et al. study [36], all-E-PTF and three Z-PTF isomers were detected in the plasma of an individual of Asian ethnicity 24 h after the consumption of fruits and vegetables.

Faeces chromatograms also showed a single peak at 17.5 min and with a 285 nm spectra and was identified as PT, similar to what was reported in a faeces study with a peak at 13.5 min using a methanol/TBME/water mobile phase [37]. In contrast, three peaks were identified as PTF in faeces but only one peak was observed in serum. These peaks eluted at 20, 22.5 and 24 min, and their maximum absorption was at 330, 347 and 363 nm. The one that appeared first was the most frequent and abundant. Similarly, the Stinco et al. [37] study detected five different PTF isomers in faeces samples attributable to the intake of vegetables purees containing tomato between minutes 16 and 17 (with spectra at 332, 348 and 367 nm), using a C30 column and ethanol (solvent A), methyl-tert-butyl ether (solvent B) and water (solvent C) in the mobile phase.

PT and PTF serum concentrations (0.158 and 0.054 µmol/L (8.62 and 2.93 μg/dL), respectively, were higher for PT and lower for PTF than those described in the literature that range from 2 to nearly 8 μg/dL (0.035–0.145 µM) for PT and from about 8 to 24 μg/dL for PTF (0.145–0.440 µmol/L) [5,38,39,40]. In our studies, dietary concentration of PT (this and other study on Spanish population [2]) and PT concentration in faeces [18] were higher than PTF concentrations. Higher PT than PTF serum concentrations were also observed, which is the opposite of what was described in other studies that show PTF serum concentrations to be higher [5,38,39,40] or similar [10] to PT. These discrepancies could be partially due to the very low number of subjects in the other studies (between 3 and 36).

The PT and PTF serum concentrations in this study can be compared to the average value of the six major serum carotenoids reported in a recent review compiling data from several countries [4]: α-carotene (100 nmol/L), β-carotene (500 nmol/L), lycopene (600 nmol/L), β-cryptoxanthin (230 nmol/L), and lutein and zeaxanthin (330 nmol/L). The PTF serum concentration (54 nmol/L) was lower than those major carotenoids, but the PT concentration (158 nmol/L) was higher than that of α-carotene.

PT and PTF dietary intake and serum concentrations were not affected by the fruit and vegetable (rich in lutein) dietary intervention, even though the fruit intervention group included oranges, a food with higher PT and PTF content [2,7]. A 19-week cross-over dietary intervention study on 23 volunteers with watermelon juice and tomato juice showed a slight increase in PT concentration in blood but not for PTF when watermelon juice was consumed [9]; however, a two-fold in PT and PTF concentration was observed after tomato juice intake. This study showed that PT was bioavailable from both tomato and watermelon juices, but particularly from tomato juice. Moreover, in the case of dietary intervention, a decrease in faeces elimination of PT and PTF (isomer 3) were observed in the fruit group, likely due to the decrease in carrot consumption in this group, a food that contributes significant amounts of PT and PTF. It also showed that the decrease was significant in participants who initially had low serum values. With an aim to increase serum levels, intestinal absorption of PT and PTF may be greater, thus reducing elimination in faeces and increasing serum levels. However, no serum effects were observed 4 weeks after the intervention. The lack of response in PT and PTF serum concentrations after the dietary intervention could be due to the short duration of dietary intervention (4 weeks) or insufficient PT and PTF dietary intake (in fact, there was no increase in their dietary intake as a proportion of the whole diet) or due to possible storage of these carotenoids in other tissues.

## 5. Conclusions

PT showed higher concentrations than PTF in serum, faeces and in the dietary intake. Carrot and tomato were the major dietary contributors of these carotenoids in the diets of Spanish adults in this study. The dietary intervention with lutein-rich fruits and vegetables did not cause significant variations in the PT and PTF intake or serum concentrations. Data on PT and PTF concentrations in serum and faeces and dietary intake in healthy adults, compared to the major carotenoids being studied so far in the context of diet and health highlight the need for further research on the function of these colourless carotenoids in humans.

## Figures and Tables

**Table 1:** Phytoene and phytofluene concentrations in serum (µmol/L), faeces μg/g (dry weight) and dietary intake from major contributors to their dietary intake (µg/day) in the observational study (*n* = 101) (mean ± SD [median]).

**Serum Concentration (µmol/L)**	
Phytoene	0.166 ± 0.067 (0.154)
Phytofluene	0.058 ± 0.039 (0.044)
**Faecal Concentration (μg/g d.w.) ^1^**	
Phytoene	16.43 ± 22.04 (11.4)
Phytofluene	5.53 ± 8.11 (3.0)
**Phytoene Dietary Intake From (µg/day) ^2^**	
Apricot	126.1 ± 481.8 (0.0)
Carrot	1424.6 ± 2069.9 (697.3)
Orange	31.5 ± 62.8 (0.0)
Orange juice	26.0 ± 57.1 (0.0)
Tomato	1135.3 ± 1195.7 (894.3)
Tomato fried	0.0 ± 0.0 (0.0)
Tomato pure	21.0 ± 65.6 (0.0)
Tomato juice	26.3 ± 263.9 (0.0)
Watermelon	35.0 ± 143.2 (0.0)
**Phytofluene Dietary Intake From (µg/day) ^1^**	
Apricot	27.6 ± 105.3 (0.0)
Carrot	333.6 ± 484.7 (163.3)
Orange	14.4 ± 28.7 (0.0)
Orange juice	8.5 ± 18.7 (0.0)
Tomato	220.8 ± 232.5 (173.9)
Tomato fried	76.0 ± 149.4 (0.0)
Tomato pure	11.2 ± 34.9 (0.0)
Tomato juice	11.6 ± 117.0 (0.0)
Watermelon	13.4 ± 54.7 (0.0)

^1^ d.w.: dry weight. PT and PTF median values was published elsewhere [18]; ^2^ PT and PTF dietary intake were 2.7 and 0.61, respectively [18].

**Table 2:** Phytoene and phytofluene concentrations in serum (µmol/L), faeces (μg/g dry weight) and dietary intake from major contributors to their dietary intake (µg/day) in the intervention study (Mean ± SD [median]).

	Fruit Group (*n* = 14)		Vegetable Group (*n* = 15)	
	Basal	Final	Basal	Final
Serum Concentration				
Phytoene	0.119 ± 0.035 (0.107)	0.110 ± 0.025 (0.109)	0.143 ± 0.082 (0.116)	0.138 ± 0.074 (0.110)
Phytofluene	0.036 ± 0.027 (0.026)	0.034 ± 0.018 (0.027)	0.043 ± 0.030 (0.038)	0.049 ± 0.031 (0.038)
Faecal Concentration				
Phytoene	23.27 ± 12.57 (24.47) ^a^	14.48 ± 8.69 (10.91) ^a,^*	19.97 ± 11.29 (19.13)	23.37 ± 11.56 (19.49) *
Total phytofluene	10.98 ± 6.96 (13.34)	7.39 ± 4.20 (5.83)	8.39 ± 5.82 (7.53)	10.35 ± 5.44 (8.87)
Isomer 1	6.61 ± 4.73 (7.27)	4.23 ± 2.96 (3.41)	4.41 ± 3.52 (4.26)	5.49 ± 3.49 (4.33)
Isomer 2	3.63 ± 2.19 (4.03)	2.70 ± 1.19 (2.55)	3.07 ± 2.18 (2.95)	3.79 ± 1.76 (3.30)
Isomer 3	0.73 ± 0.50 (0.94) ^a^	0.47 ± 0.27 (0.47) ^a*^	0.92 ± 0.66 (0.97)	1.07 ± 0.75 (1.01) *
Dietary Intake				
Phytoene	2229.8 ± 2212.8 (1657.7)	1856.7 ± 1505.9 (1675.1)	3037.2 ± 1784.8 (2837.8)	2281.7± 1542.3 (2043.2)
Phytofluene	547.4 ± 491.1 (437.7)	459.5 ± 305.5 (440.4)	797.4 ± 438.7 (640.0)	518.9 ± 344.8 (412.5)
PT Dietary Intake From				
Apricot	0.0 ± 0.0 (0.0)	0.0 ± 0.0 (0.0)	28.7 ± 111.3(0.0)	488.5 ± 1078.5 (0.0)
Carrot	1172.1 ± 2157.9 (526.7) ^a^	479.4 ± 806.1 (0.0) ^a^	1475.1 ± 2068.9 (632.0)	621.8 ± 1106.6 (0.0)
Orange	93.0 ± 80.0 (89.7)	126.3 ± 52.2 (120.5)	32.4 ± 60.7 (0.0)	20.5 ± 38.0 (0.0)
Orange juice	0.0 ± 0.0 (0.0) *	5.8 ± 21.8 (0.0) *	29.9 ± 58.6 (0.0) ^a,^*	0.0 ± 0.0 (0.0) ^a,^*
Tomato	935.3 ± 953.2 (531.2)	1212.3 ± 985.4 (1095.4)	1406.1 ± 1313.2 (1169.2)	970.1 ± 759.1 (797.6)
Tomato fried	0.0 ± 0.0 (0.0)	0.0 ± 0.0 (0.0)	0.0 ± 0.0 (0.0)	0.0 ± 0.0 (0.0)
Tomato pure	20.5 ± 55.0 (0.0)	20.1 ± 75.3 (0.0)	12.5 ± 33.0 (0.0)	12.5 ± 48.5 (0.0)
Tomato juice	0.0 ± 0.0 (0.0)	0.0 ± 0.0 (0.0)	0.0 ± 0.0 (0.0)	0.0 ± 0.0 (0.0)
Watermelon	0.0 ± 0.0 (0.0)	2.9 ± 10.8 (0.0)	40.4 ± 91.4 (0.0)	88.2 ± 146.9 (0.0)
PTF Dietary Intake From				
Apricot	0.0 ± 0.0 (0.0)	0.0 ± 0.0 (0.0)	6.3 ± 24.3 (0.0)	106.8 ± 235.8 (0.0)
Carrot	274.5 ± 505.3 (123.4) ^a^	112.3 ± 188.8 (0.0) ^a^	345.4 ± 484.5 (148.0)	145.6 ± 259.1 (0.0)
Orange	42.4 ± 36.5 (40.9.)	57.6 ± 23.8 (55.0) *	14.8 ± 27.7 (0.0)	9.3 ± 17.4 (0.0) *
Orange juice	0.0 ± 0.0 (0.0) *	1.9 ± 7.1 (0.0)	9.8 ± 19.2 (0.0) ^a,^*	0.0 ± 0.0 (0.0) ^a^
Tomato	181.9 ± 185.4 (103.3)	235.7 ± 191.6 (213.0)	273.4 ± 255.4 (227.4)	188.7 ± 147.6 (155.1)
Tomato fried	28.7 ± 49.8 (0.0)	36.1 ± 52.2 (0.0) *	112.3 ± 178.2 (0.0) ^a^	8.8 ± 34.0 (0.0) ^a,^*
Tomato pure	10.9 ± 29.2 (0.0)	10.7 ± 40.1 (0.0)	6.7 ± 17.6 (0.0)	6.7 ± 25.8 (0.0)
Tomato juice	0.0 ± 0.0 (0.0)	0.0 ± 0.0 (0.0)	0.0 ± 0.0 (0.0)	0.0 ± 0.0 (0.0)
Watermelon	0.0 ± 0.0 (0.0)	1.1 ± 4.1 (0.0)	15.4 ± 34.9 (0.0)	33.7 ± 56.1 (0.0)

^a^ Significant differences between final and basal data; * Significant differences between fruit and vegetable groups.

## References

[1] Engelmann N.J., Clinton S.K., Erdman J.W. (2011). Nutritional aspects of phytoene and phytofluene, carotenoid precursors to lycopene. Adv. Nutr..

[2] Olmedilla-Alonso B., Benítez-González A.M., Estévez-Santiago R., Mapelli-Brahm P., Stinco C.M., Meléndez-Martínez A.J. (2021). Assessment of Food Sources and the Intake of the Colourless Carotenoids Phytoene and Phytofluene in Spain. Nutrients.

[3] Biehler E., Alkerwi A., Hoffmann L., Krause E., Guillaume M., Lair M., Bohn T. (2012). Contribution of violaxanthin, neoxanthin, phytoene and phytofluene to total carotenoid intake: Assessment in Luxembourg. J. Food Compos. Anal..

[4] Böhm V., Lietz G., Olmedilla-Alonso B., Phelan D., Reboul E., Bánati D., Borel P., Corte-Real J., de Lera A.R., Desmarchelier C. (2021). From carotenoid intake to carotenoid blood and tissue concentrations—implications for dietary intake recommendations. Nutr. Rev..

[5] Porrini M., Riso P., Brusamolino A., Berti C., Guarnieri S., Visioli F. (2005). Daily intake of a formulated tomato drink affects carotenoid plasma and lymphocyte concentrations and improves cellular antioxidant protection. Br. J. Nutr..

[6] Campbell J.K., Engelmann N.J., Lila M.A., Erdman J.W. (2007). Phytoene, phytofluene, and lycopene from tomato powder differentially accumulate in tissues of male Fisher 344 rats. Nutr. Res..

[7] Meléndez-Martínez A.J., Mapelli-Brahm P., Benítez-González A., Stinco C.M. (2015). A comprehensive review on the colorless carotenoids phytoene and phytofluene. Arch. Biochem. Biophys..

[8] Richelle M., Bortlik K., Liardet S., Hager C., Lambelet P., Baur M., Applegate L.A., Offord E.A. (2002). A food-based formulation provides lycopene with the same bioavailability to humans as that from tomato paste. J. Nutr..

[9] Edwards A.J., Vinyard B.T., Wiley E.R., Brown E.D., Collins J.K., Perkins-Veazie P., Baker R.A., Clevidence B.A. (2003). Consumption of watermelon juice increases plasma concentrations of lycopene and beta-carotene in humans. J. Nutr..

[10] Müller H., Bub A., Watzl B., Rechkemmer G. (1999). Plasma concentrations of carotenoids in healthy volunteers after intervention with carotenoid-rich foods. Eur. J. Nutr..

[11] Fuller B., Smith D., Howerton A., Kern D. (2006). Anti-inflammatory effects of CoQ10 and colorless carotenoids. J. Cosmet. Dermatol..

[12] Nara E., Hayashi H., Kotake M., Miyashita K., Nagao A. (2001). Acyclic carotenoids and their oxidation mixtures inhibit the growth of HL-60 human promyelocytic leukemia cells. Nutr. Cancer.

[13] Hirsch K., Atzmon A., Danilenko M., Levy J., Sharoni Y. (2007). Lycopene and other carotenoids inhibit estrogenic activity of 17beta-estradiol and genistein in cancer cells. Breast Cancer Res. Treat..

[14] Ben-Dor A., Steiner M., Gheber L., Danilenko M., Dubi N., Linnewiel K., Zick A., Sharoni Y., Levy J. (2005). Carotenoids activate the antioxidant response element transcription system. Mol. Cancer Ther..

[15] Mapelli-Brahm P., Corte-Real J., Meléndez-Martínez A.J., Bohn T. (2017). Bioaccessibility of phytoene and phytofluene is superior to other carotenoids from selected fruit and vegetable juices. Food Chem..

[16] Meléndez-Martínez A.J., Mandić A.I., Bantis F., Böhm V., Borge G.I.A., Brnčić M., Bysted A., Cano M.P., Dias M.G., Elgersma A. (2022). A comprehensive review on carotenoids in foods and feeds: Status quo, applications, patents, and research needs. Crit. Rev. Food Sci. Nutr..

[17] Hsu B.Y., Pu Y.S., Inbaraj B.S., Chen B.H. (2012). An improved high performance liquid chromatography-diode array detection-mass spectrometry method for determination of carotenoids and their precursors phytoene and phytofluene in human serum. J. Chromatogr. B Analyt. Technol. Biomed. Life Sci..

[18] Rodríguez-Rodríguez E., Beltrán-de-Miguel B., Samaniego-Aguilar K.X., Sánchez-Prieto M., Estévez-Santiago R., Olmedilla-Alonso B. (2020). Extraction and Analysis by HPLC-DAD of Carotenoids in Human Faeces from Spanish Adults. Antioxid. (Basel).

[19] Olmedilla-Alonso B., Rodríguez-Rodríguez E., Beltrán-De-Miguel B., Estévez-Santiago R., Sánchez-Prieto M. (2021). Predictors of macular pigment and contrast threshold in normolipemic subjects aged 45–65. PLoS ONE.

[20] Olmedilla-Alonso B., Rodríguez-Rodríguez E., Beltrán-de-Miguel B., Sánchez-Prieto M., Estévez-Santiago R. (2021). Changes in Lutein Status Markers (Serum and Faecal Concentrations, Macular Pigment) in Response to a Lutein-Rich Fruit or Vegetable (Three Pieces/Day) Dietary Intervention in Normolipemic Subjects. Nutrients.

[21] Dias M.G., Olmedilla-Alonso B., Hornero-Méndez D., Mercadante A.Z., Osorio C., Vargas-Murga L., Meléndez-Martínez A.J. (2018). Comprehensive database of carotenoid contents in ibero-american foods. A valuable tool in the context of functional foods and the establishment of recommended intakes of bioactives. J. Agric. Food Chem..

[22] Rodríguez-Rodríguez E., Sánchez-Prieto M., Olmedilla-Alonso B. (2020). Assessment of carotenoid concentrations in red peppers (Capsicum annuum) under domestic refrigeration for three weeks as determined by HPLC-DAD. Food Chem. X.

[23] de Rosso V.V., Mercadante A.Z. (2007). Identification and quantification of carotenoids, by HPLC-PDA-MS/MS, from Amazonian fruits. J. Agric. Food Chem..

[24] Estévez-Santiago R., Beltrán-de-Miguel B., Cuadrado-Vives C., Olmedilla-Alonso B. (2013). Software application for the calculation of dietary intake of individual carotenoids and of its contribution to vitamin A intake. Nutr. Hosp..

[25] Meléndez-Martínez A.J., Mapelli-Brahm P., Stinco C.M. (2018). The colourless carotenoids phytoene and phytofluene: From dietary sources to their usefulness for the functional foods and nutricosmetics industries. J. Food Compos. Anal..

[26] Maiani G., Castón M.J., Catasta G., Toti E., Cambrodón I.G., Bysted A., Granado-Lorencio F., Olmedilla-Alonso B., Knuthsen P., Valoti M. (2009). Carotenoids: Actual knowledge on food sources, intakes, stability and bioavailability and their protective role in humans. Mol. Nutr. Food Res..

[27] Meléndez-Martínez A.J., Fraser P.D., Bramley P.M. (2012). Accumulation of health promoting phytochemicals in wild relatives of tomato and their contribution to in vitro antioxidant activity. Phytochemistry.

[28] Olmedilla-Alonso B., Beltrán-de-Miguel B., Estévez-Santiago R., Cuadrado-Vives C. (2014). Markers of lutein and zeaxanthin status in two age groups of men and women: Dietary intake, serum concentrations, lipid profile and macular pigment optical density. Nutr. J..

[29] Beltrán-de-Miguel B., Estévez-Santiago R., Olmedilla-Alonso B. (2015). Assessment of dietary vitamin A intake (retinol, α-carotene, β-carotene, β-cryptoxanthin) and its sources in the National Survey of Dietary Intake in Spain (2009–2010). Int. J. Food Sci. Nutr..

[30] Estévez-Santiago R., Beltrán-de-Miguel B., Olmedilla-Alonso B. (2016). Assessment of dietary lutein, zeaxanthin and lycopene intakes and sources in the Spanish survey of dietary intake (2009–2010). Int. J. Food Sci. Nutr..

[31] Than A., Bramley P.M., Davies B.H., Rees A.F. (1972). Stereochemistry of phytoene. Phytochemistry.

[32] Mapelli-Brahm P., Desmarchelier C., Margier M., Reboul E., Meléndez Martínez A.J., Borel P. (2018). Phytoene and Phytofluene Isolated from a Tomato Extract are Readily Incorporated in Mixed Micelles and Absorbed by Caco-2 Cells, as Compared to Lycopene, and SR-BI is Involved in their Cellular Uptake. Mol. Nutr. Food Res..

[33] Yu J., Gleize B., Zhang L., Caris-Veyrat C., Renard C.M.G.C. (2020). Impact of onions in tomato-based sauces on isomerization and bioaccessibility of colorless carotenes: Phytoene and phytofluene. Food Funct..

[34] Meléndez-Martínez A.J., Paulino M., Stinco C.M., Mapelli-Brahm P., Wang X.D. (2014). Study of the time-course of cis/trans (Z/E) isomerization of lycopene, phytoene, and phytofluene from tomato. J. Agric. Food Chem..

[35] de Rosso V.V., Mercadante A.Z. (2007). HPLC-PDA-MS/MS of anthocyanins and carotenoids from dovyalis and tamarillo fruits. J. Agric. Food Chem..

[36] Melendez-Martinez A.J., Stinco C.M., Liu C., Wang X.D. (2013). A simple HPLC method for the comprehensive analysis of cis/trans (Z/E) geometrical isomers of carotenoids for nutritional studies. Food Chem..

[37] Stinco C.M., Benítez-González A.M., Meléndez-Martínez A.J., Hernanz D., Vicario I.M. (2019). Simultaneous determination of dietary isoprenoids (carotenoids, chlorophylls and tocopherols) in human faeces by Rapid Resolution Liquid Chromatography. J. Chromatogr. A.

[38] Khachik F., Spangler C.J., Smith J.C., Canfield L.M., Steck A., Pfander H. (1997). Identification, quantification, and relative concentrations of carotenoids and their metabolites in human milk and serum. Anal. Chem..

[39] Paetau I., Khachik F., Brown E.D., Beecher G.R., Kramer T.R., Chittams J., Clevidence B.A. (1998). Chronic ingestion of lycopene-rich tomato juice or lycopene supplements significantly increases plasma concentrations of lycopene and related tomato carotenoids in humans. Am. J. Clin. Nutr..

[40] Aust O., Stahl W., Sies H., Tronnier H., Heinrich U. (2005). Supplementation with tomato-based products increases lycopene, phytofluene, and phytoene levels in human serum and protects against UV-light-induced erythema. Int. J. Vitam. Nutr. Res..

